# Getting value from the waste: recombinant production of a sweet protein by *Lactococcus lactis* grown on cheese whey

**DOI:** 10.1186/s12934-018-0974-z

**Published:** 2018-08-15

**Authors:** Mohamed Boumaiza, Andrea Colarusso, Ermenegilda Parrilli, Elena Garcia-Fruitós, Angela Casillo, Anna Arís, Maria Michela Corsaro, Delia Picone, Serena Leone, Maria Luisa Tutino

**Affiliations:** 1 Department of Chemical Sciences, University of Naples Federico II, via Cintia, 80126 Naples, Italy; 2 Department of Ruminant Production, Institut de Recerca i Tecnologia Agroalimentàries (IRTA), 08140 Caldes de Montbui, Spain

**Keywords:** *Lactococcus lactis*, MNEI, Nisin controlled expression system, Cheese whey, GRAS, Bioconversions, Recombinant proteins

## Abstract

**Background:**

Recent biotechnological advancements have allowed for the adoption of *Lactococcus lactis*, a typical component of starter cultures used in food industry, as the host for the production of food-grade recombinant targets. Among several advantages, *L. lactis* has the important feature of growing on lactose, the main carbohydrate in milk and a majoritarian component of dairy wastes, such as cheese whey.

**Results:**

We have used recombinant *L. lactis* NZ9000 carrying the nisin inducible pNZ8148 vector to produce MNEI, a small sweet protein derived from monellin, with potential for food industry applications as a high intensity sweetener. We have been able to sustain this production using a medium based on the cheese whey from the production of ricotta cheese, with minimal pre-treatment of the waste. As a proof of concept, we have also tested these conditions for the production of MMP-9, a protein that had been previously successfully obtained from *L. lactis* cultures in standard growth conditions.

**Conclusions:**

Other than presenting a new system for the recombinant production of MNEI, more compliant with its potential applications in food industry, our results introduce a strategy to valorize dairy effluents through the synthesis of high added value recombinant proteins. Interestingly, the possibility of using this whey-derived medium relied greatly on the choice of the appropriate codon usage for the target gene. In fact, when a gene optimized for *L. lactis* was used, the production of MNEI proceeded with good yields. On the other hand, when an *E. coli* optimized gene was employed, protein synthesis was greatly reduced, to the point of being completely abated in the cheese whey-based medium. The production of MMP-9 was comparable to what observed in the reference conditions.

**Electronic supplementary material:**

The online version of this article (10.1186/s12934-018-0974-z) contains supplementary material, which is available to authorized users.

## Background

Lactic acid bacteria (LAB) are traditional components of starter preparations and have been used for centuries in the manufacturing of fermented food [1]. More recently, they have attracted much attention for their biotechnological potential, finding use in a variety of applications. *Lactococcus lactis* (*L. lactis*), a prototypical member of this family, is a gram-positive, non-sporulating, facultative anaerobic bacterium and a common gut colonizer, which has been used for centuries by the cheese-making industry, thus receiving the Generally Recognized As Safe (GRAS) status [2, 3]. Outside food industry, *L. lactis* has been successfully exploited for its metabolic machinery, to produce and accumulate high value chemicals, such as ethanol, l-lactate, diacetyl, acetaldehyde, but also l-alanine, mannitol and other sweeteners and group B vitamins [3, 4]. Much of the biotechnological advances that have taken place for *L. lactis* own to the achievement of complete genome sequencing of a few strains [5, 6], which has allowed for metabolic engineering manipulations, aimed at favoring the production of specific metabolites of industrial interest [4, 7–10]. In this respect, it is noteworthy the production of bacteriocins, antimicrobial peptides with a number of applications, ranging from food-preservation to anti-biofilm activity in clinical set-ups [11–13].

Together with genome sequencing, the discovery and development of both constitutive and inducible expression systems for *L. lactis* has favored its development as a cell factory for the production of recombinant proteins [2, 14, 15]. Being a food-grade microorganism, devoid of endotoxins, *L. lactis* is very convenient for the heterologous production, both intracellular and secreted, of a number of therapeutics and vaccines, recently reviewed by Song et al. in [3].

The greatest boost in the use of *L. lactis* for the production of heterologous protein has been the development of the nisin controlled gene expression (NICE) system [16], which has been used in combination with the nisin-negative NZ9000 strain and its derivatives for the production of numerous recombinant protein targets [17].

In parallel with their use as molecular biology tools, *L. lactis* and other LAB have found space in the field of bioconversions, where their ability to digest the components of industrial effluents has been coupled to the production of added value chemicals [18–25]. In the case of LAB, their ability to digest lactose has been functional to the possibility of using them for the valorization of dairy wastes. Cheese whey (CW), the serum portion of milk that survives the process of cheese making, is in fact one of the most polluting by-products of dairy industry and its disposal imposes a considerable economic burden on cheese producers, due to its high biological and chemical oxygen demand [26]. The composition of CW varies according to its origin, but, in general, it is a nutritious mixture, containing roughly half the solids of raw milk. In particular, most of the lactose (that survives protein agglutination), roughly 20% of the original milk proteins, some vitamins and minerals are still present in CW, which is in fact often used as feeding for livestock [27–29]. In addition to its use “as is”, strategies for the valorization of CW have been proposed, often employing microbes to convert it in biomass or added value compounds, like ethanol, organic acids or bacteriocins [18, 20, 28, 30–32]. Nonetheless, to our knowledge, this material has never been used to sustain the production of high value recombinant proteins.

We here describe the use of the CW resulting from the making of mozzarella and ricotta cheese, to sustain the growth of *L. lactis* NZ9000 and the heterologous production of the small (~ 12 kDa), globular protein MNEI. MNEI is a single chain derivative of the plant (*Dioscoreophyllum cumminsii*) protein monellin [33, 34] and has received much attention for its high intensity sweetness. Due to this potential industrial interest, several biotechnological strategies have been devised to produce it, exploiting a variety of host systems [35, 36]. MNEI is one of the best characterized members of the sweet proteins family and it has been object of protein engineering to improve its taste profile and physicochemical characteristics, trying to meet the needs of the food and beverage industry [37–43]. In light of its potential applications in food preparations, the optimization of a food-grade production system becomes particularly important. The possibility of coupling such system to the valorization of a largely available industrial by-product makes our strategy particularly appealing. Moreover, we demonstrate that these growth conditions can be successfully used to produce other recombinant proteins in *L. lactis* strains. In particular, we show that the expression levels of the previously characterized protein MMP-9 in *L. lactis* [44, 45] are comparable in the CW-based medium and in the reference conditions in rich medium.

## Results

### Expression of MNEI in *L. lactis* NZ9000

**Fig. 1:**
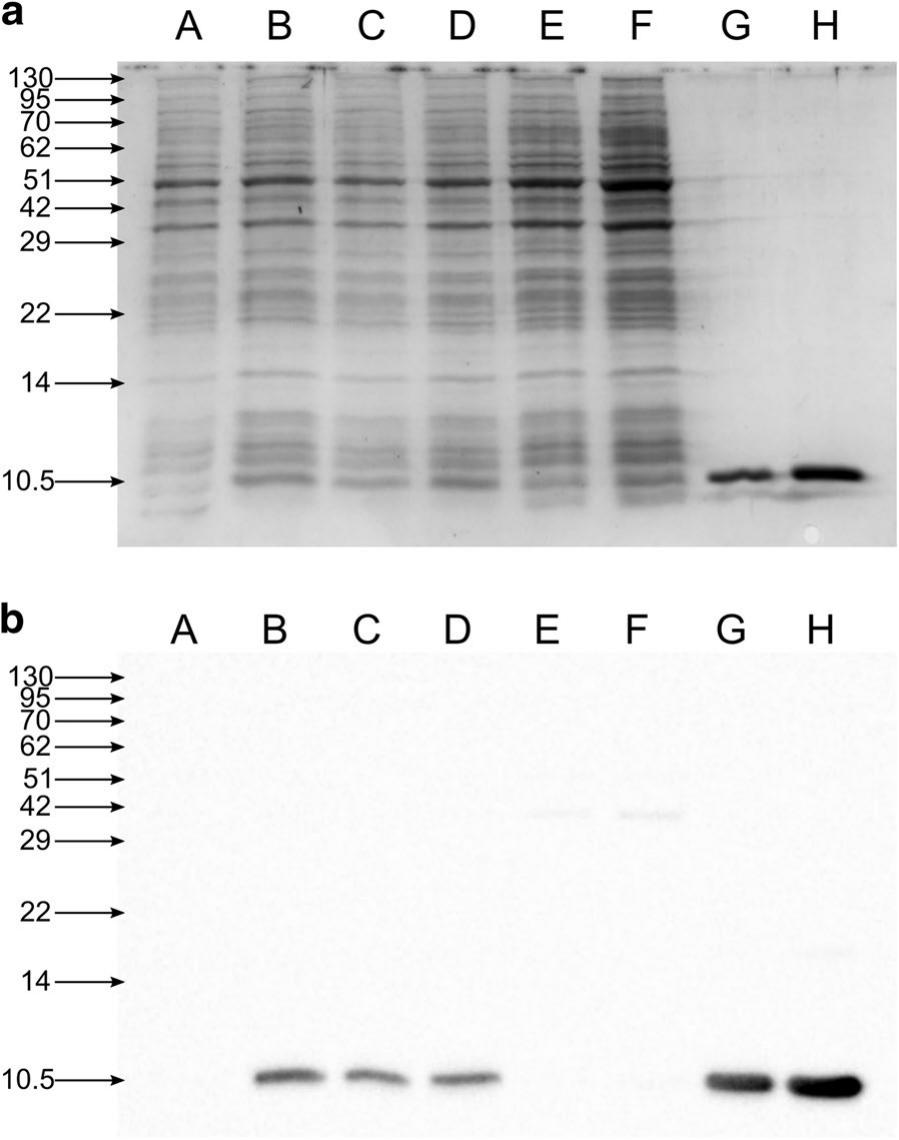
Production of recombinant MNEI in G-M17 medium. Coomassie-stained SDS-PAGE (**a**) and western blot (**b**) of the total protein extract (10 μg) from *L. lactis* NZ9000 carrying the pNZ8148-*MNEI*-*ll* vector (A–D) or the empty pNZ8148 vector (E–F). A: no induction; B: 2 h post-induction; C: 4 h post induction; D: 16 h post induction; E: no induction; F: 4 h post induction; G: MNEI standard, 200 ng; H: MNEI standard, 500 ng

The synthetic genes coding for MNEI with optimized codon usage for *E. coli* (*MNEI*-*ec*) and *L. lactis* (*MNEI*-*ll*) were cloned into the pNZ8148 vector. This was motivated by the fact that previous studies aimed at the production of brazzein, another sweet protein, in *L. lactis* had shown the counterintuitive result of greater production yields when employing an *E. coli* optimized gene [46–48]. In our case, the two synthetic genes differed for their GC content (49% and 37% for *MNEI*-*ec* and *MNEI*-*ll*, respectively) and their sequence alignment is reported in Additional file 1: Figure S1. Unlike the previous results for brazzein, when *L. lactis* NZ9000 was transformed with the vector containing the *MNEI*-*ec* gene, negligible protein production was observed (Additional file 2: Figure S2). Conversely, the system carrying the pNZ8148-*MNEI*-*ll* vector proved to be quite efficient: Fig. 1 shows the Coomassie stained SDS-PAGE and the western blot of 10 μg of total protein extracts from recombinant *L. lactis* producing MNEI. Densitometric quantification of the blot provided an estimate of the yield ~ 140 ng MNEI in 10 μg of total protein extract, corresponding to ~ 0.40 mg of protein per liter of culture medium, which remains quite stable throughout the fermentation, based on the intensity of the bands corresponding to 2, 4 and 16 h post-induction in comparison to purified protein samples.

### Growth of *L. lactis* NZ9000 on cheese whey based media

The above experiments were all carried out in the conventional growth conditions for *L. lactis*, namely static cultures in M17 rich medium supplemented with 0.5% glucose (G-M17) at 30 °C. We then moved to comparing the microbial growth on CW. We used the CW obtained as by-product of the manufacturing of ricotta, a typical fresh cheese from Campania, Italy. Ricotta is produced by heating at 90 °C the whey resulting from the production of “Mozzarella di Bufala Campana” and collecting the flocculating suspension. The residual watery portion constitutes the CW employed in this study. Upon delivery to our lab, all CW samples contained variable amounts of particulate in suspension, formed during the cooling down of the exhausted whey. Several treatments have been proposed as preliminary workups when CW is used as a raw material for biotechnological applications, and these range from total deproteinization of CW, to treatment with proteolytic enzymes, to supplementation with other nutrients such as peptone and yeast extract [20, 21]. Since we were trying to set up a scalable protocol, ideally suitable to work on the large amounts of CW produced by a dairy industry, we decided to limit the steps prior to inoculation of *L. lactis* to pH adjustment to 6.8, sterilization by autoclave and clarification by centrifugation of CW (ac-CW). The average nutrients content of CW and ac-CW before bacterial culture is provided in Table 1. The counterintuitive increase in carbohydrate content upon sterilization might result from partial hydrolysis of lactose, the main sugar of milk, to glucose and galactose. This would provide a higher response to the phenol assay used to quantify reducing carbohydrates. Despite pH adjustment had been performed, the lack of buffering capacity of untreated CW resulted in a drop in pH after sterilization, to ~ 6.2. Nonetheless, given the tolerance of LAB for acidic pHs, no further adjustment was performed. The medium was also supplemented with a catalytic amount (0.05%) of yeast extract as a source of vitamins and cofactors, to improve the utilization of the nutrients, still abundant in ac-CW.

**Table 1:** Mean composition and standard deviation of the cheese whey (CW) at reception and after sterilization/clarification (ac-CW)

	CW	ac-CW
Carbohydrates (mg/mL)	27 ± 2	40 ± 5
Proteins (mg/mL)	2.4 ± 0.3	2.3 ± 0.2
Lipids (mg/mL)	12 ± 5	7 ± 3
pH	6.1 ± 0.1	6.17 ± 0.04

**Fig. 2:**
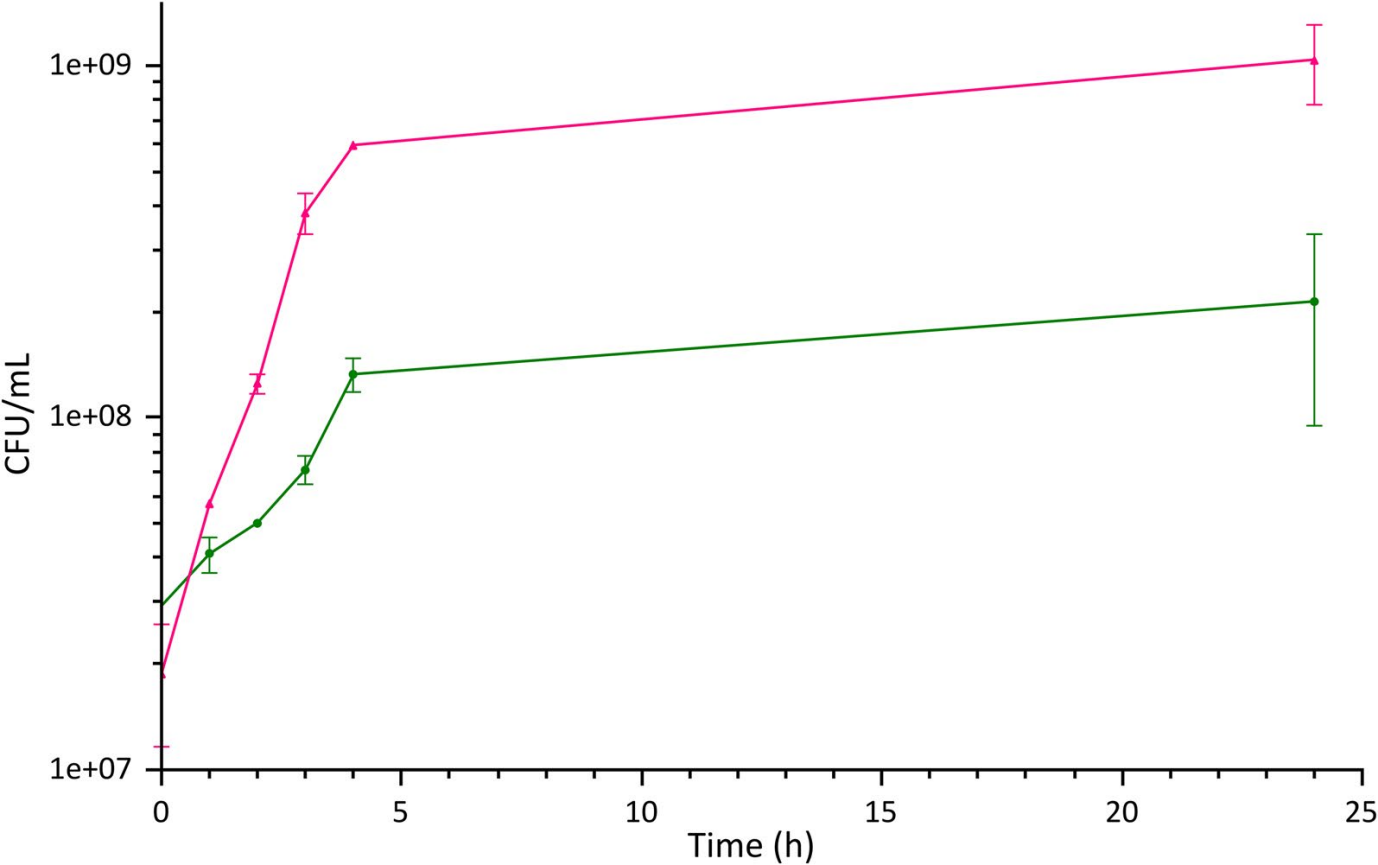
Growth curves of *L. lactis* NZ9000 in different media. The figure shows a comparison of the growth curve of *L. lactis* NZ9000 in the reference rich medium G-M17 (red curve) and on ac-CW supplemented with 0.05% yeast extract as a source of cofactors and vitamins (green curve). The final biomass in the two conditions differs by one order of magnitude

Explorative experiments showed that the growth on the CW-based medium was accompanied by a marked decrease in pH, due to the production of organic acids, paralleling what observed during the growth in G-M17 medium (Additional file 3: Figure S3). Therefore, since the proteins in CW had not been hydrolyzed, the growth in this medium was accompanied by additional protein precipitation, making it impossible to monitor biomass accumulation with turbidimetric methods (i.e. by measuring the OD600). Thus, all the growth curves were constructed measuring the CFUs/mL at different time points. Figure 2 shows the comparison of the growth curves, while Table 2 summarizes the growth parameters, for *L. lactis* NZ9000 in the standardized G-M17 medium and in the ac-CW-based medium. The marked difference in biomass at the end of the growth (2.14 × 10^8^ vs 1.04 × 10^9^ CFU/mL in ac-CW medium and G-M17, respectively) is likely the result of the significant protein deficiency of the ac-CW-based medium compared to G-M17 (2.3 vs 15.0 g/L, Additional file 4: Table S1) and of an unbalanced carbohydrates:proteins ratio (40:2.3 vs 10:15). Nonetheless, despite these suboptimal conditions, *L. lactis* NZ9000 could thrive in the CW-derived medium.

**Table 2:** Comparison of the growth parameters for *L. lactis* NZ9000 in G-M17 medium and ac-CW + 0.05% yeast extract

	Growth rate µmax (h^−1^)	Doubling time td (h)
G-M17	1.1 ± 0.2	0.6 ± 0.1
Ac-CW + 0.05% yeast extract	0.6 ± 0.1	1.2 ± 0.1

### Expression of recombinant MNEI on CW-based medium

**Fig. 3:**
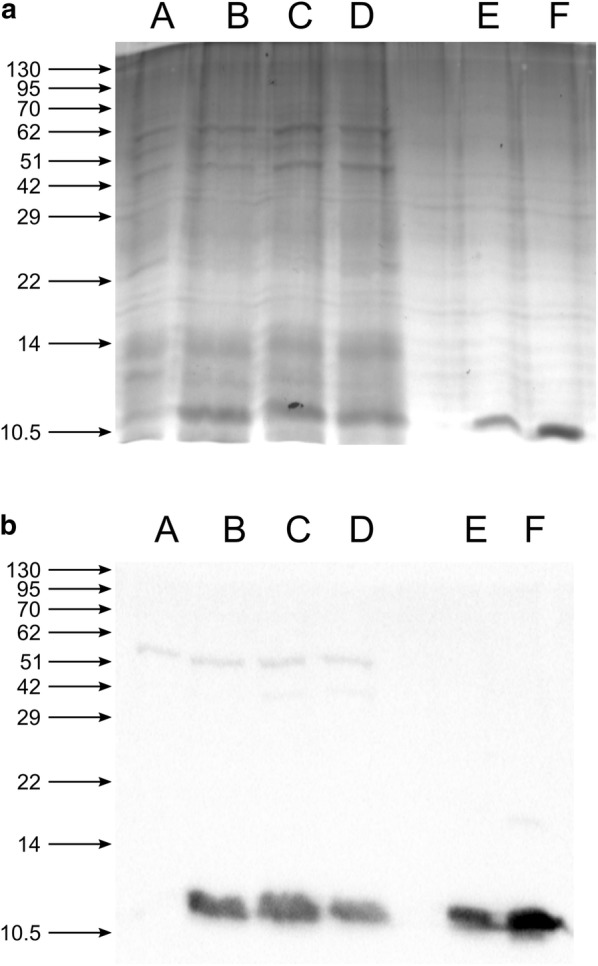
Production of recombinant MNEI in the ac-CW based medium. Coomassie-stained SDS-PAGE (**a**) and western blot (**b**) of the total protein extract (10 μg) from *L. lactis* NZ9000 carrying the pNZ8148-*MNEI*-*ll* and growing on ac-CW + 0.05% yeast extract. A: no induction; B: 2 h post-induction; C: 4 h post induction; D: 16 h post induction; E: MNEI standard, 200 ng; F: MNEI standard, 500 ng

Once we had ascertained the capacity of *L. lactis* to grow on the ac-CW-based medium, we checked if these conditions could also sustain the expression of MNEI in the recombinant strain carrying the pNZ8148-*MNEI*-*ll* or pNZ8148-*MNEI*-*ec* vector. Both strains showed comparable growth profiles to the wild type strain (not shown); therefore, in all subsequent experiments, protein synthesis was induced after 2 h, corresponding to the mid-exponential phase, with 10 ng/mL nisin and checked at different time points post-induction by Western Blot. Figure 3 shows the Coomassie-stained SDS-PAGE (A) and Western blot (B) of the total protein extracts at different times post induction. Despite the discussed reduction in biomass accumulation compared to the standard G-M17 rich medium, the yield of recombinant MNEI on the ac-CW-based medium seems higher than in the reference condition. Densitometric quantification of MNEI by western blot, in comparison with pure protein samples, allowed us to estimate a yield of 270 ng MNEI/10 μg total proteins 2 h after induction, i.e. almost twice the observed yield in G-M17 medium. The yield of protein per liter of culture medium was nonetheless comparable to that in G-M17, i.e. ~ 0.49 mg, because of the lower biomass reached in ac-CW-based growth conditions. Interestingly, when the recombinant strain carrying the pNZ8148-*MNEI*-*ec* vector was grown on the ac-CW-based medium, no protein production could be detected (Additional file 5: Figure S4).

### Expression of MMP-9 on CW-based medium

**Fig. 4:**
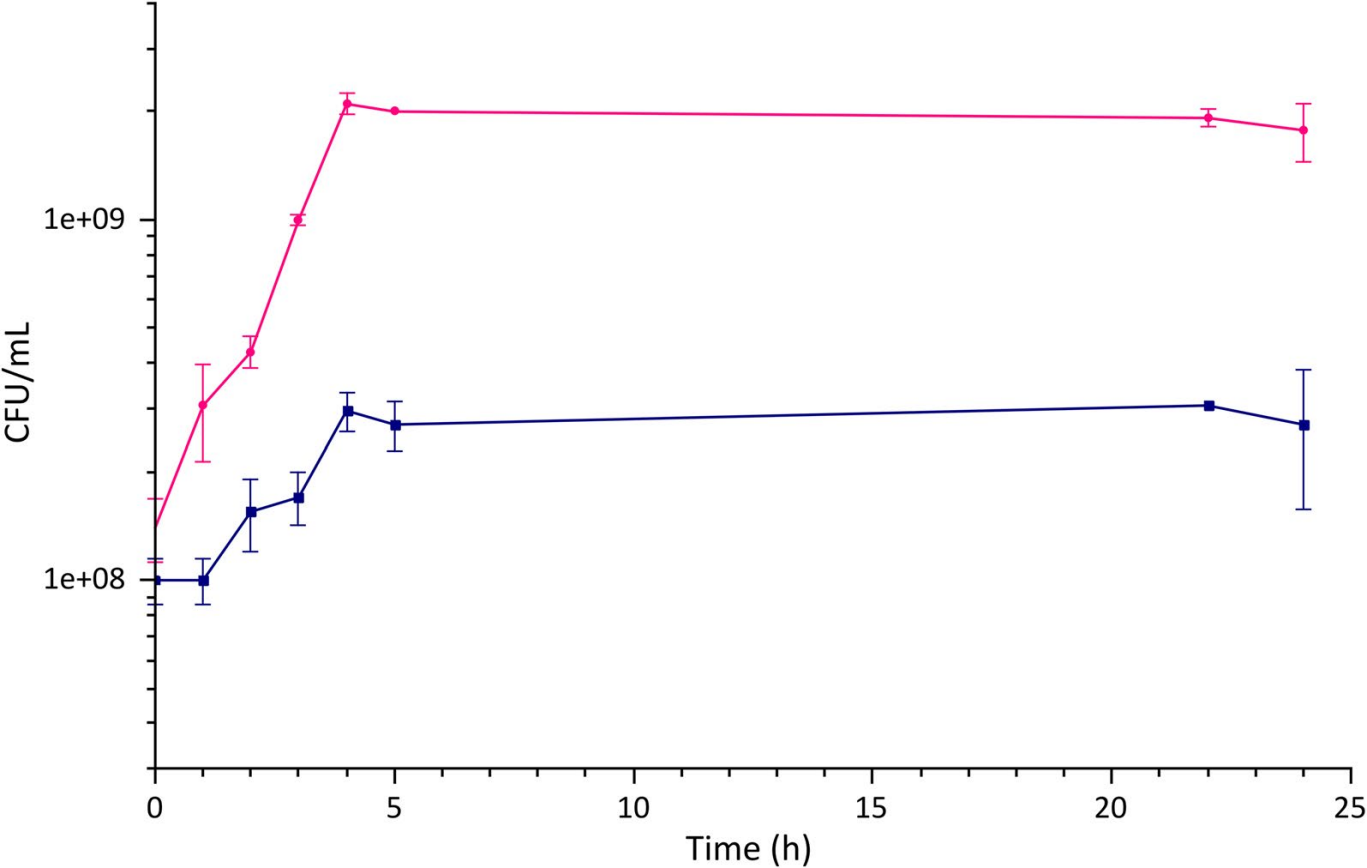
Comparison of the growth of *L. lactis* NZ9000 *clpP*^−^ *htrA*^−^ in different media. The figure shows a comparison of the growth curve of *L. lactis* NZ9000 *clpP*^−^ *htrA*^−^ carrying the pNZ8148-*MMP*-*9* vector in the reference rich medium (pink curve), with the growth on ac-CW supplemented with 0.05% yeast extract (blue curve)

**Fig. 5:**
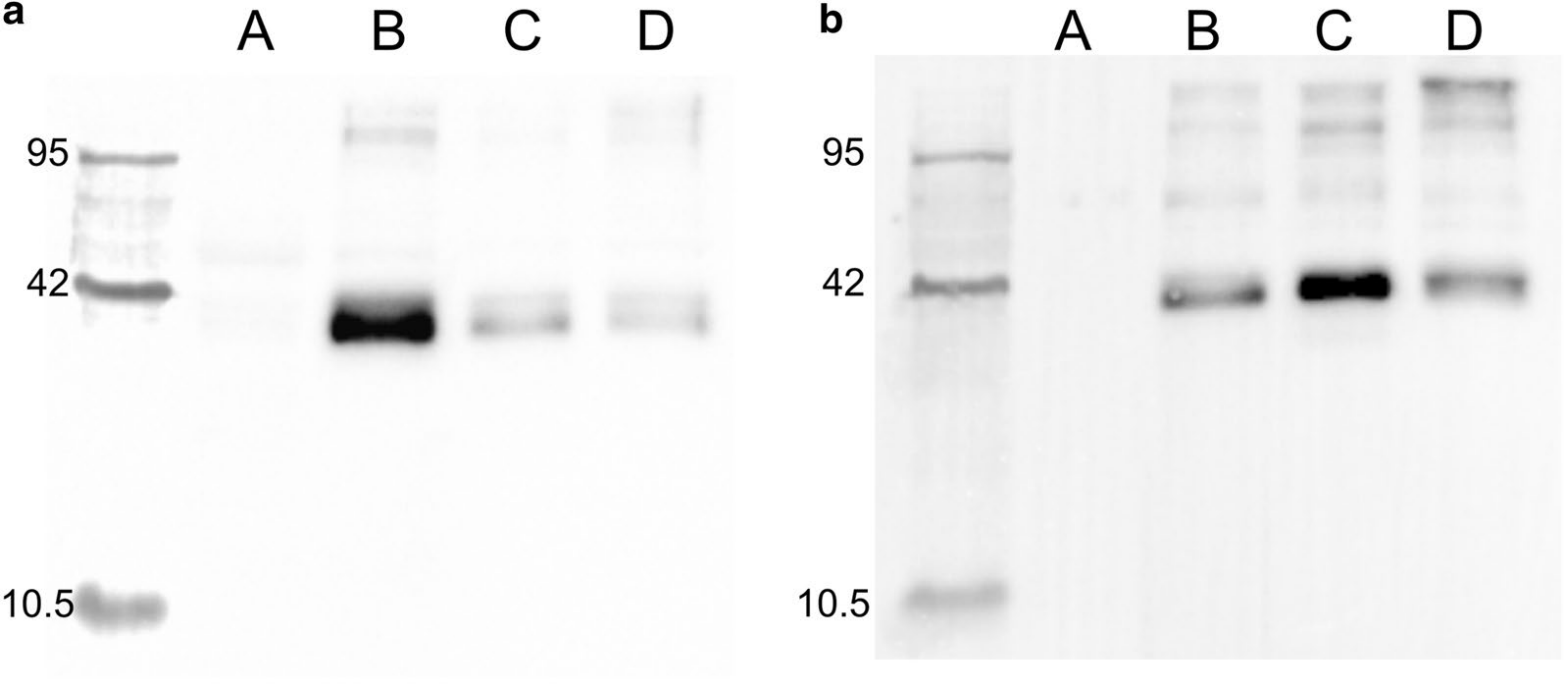
Production of recombinant MMP9 in the G-M17 and ac-CW based medium. The figure shows a comparison of the western blots of 10 μg of total protein extract of cells of *L. lactis* NZ9000 *clpP*^−^ *htrA*^−^ transformed with the pNZ8148-*MMP9* vector after culture in rich medium (**a**) or in ac-CW supplemented with 0.05% Yeast extract (**b**). A: no induction; B: 2 h post-induction; C: 4 h post induction; D: 16 h post induction. While protein expression levels seem comparable in the two cases, maximum production appears slightly delayed in the case of the CW derived medium

As a proof of concept, to demonstrate the suitability of the ac-CW-based medium to produce recombinant proteins in *L. lactis*, we tested its efficiency on a previously characterized system, namely *L. lactis* NZ9000 *clpP*^−^ *htrA*^−^ carrying the pNZ8148-*MMP*-*9* vector coding for the catalytic domain (Phe107-Pro449, ~ 40 KDa) of metalloproteinase 9 (MMP-9) [44, 45]. Figure 4 shows the comparison between the growth curves in G-M17 and in ac-CW with 0.05% yeast extract. The comparison of the growth parameters for the two conditions is provided in Table 3. Compared to the reference condition in rich medium, we observe again the substantial reduction of the final biomass accumulation. Nonetheless, when MMP-9 expression was induced, with 10 ng/mL nisin after 2 h, comparable production of recombinant protein was obtained, as visible in Fig. 5, which shows the western blots of the total protein lysates obtained at different times post-induction in the two conditions examined. Maximal accumulation of the recombinant target seems only slightly delayed compared to the reference condition.

**Table 3:** Comparison of the growth parameters for *L. lactis* NZ9000 *clpP*^−^ *htrA*^−^ (pNZ8148-*MMP*-*9*) in G-M17 medium and ac-CW + 0.05% yeast extract

	Growth rate µmax (h^−1^)	Doubling time td (h)
G-M17	0.8 ± 0.1	0.8 ± 0.1
Ac-CW + 0.05% yeast extract	0.6 ± 0.2	1.2 ± 0.2

## Discussion

The possibility of reintroducing waste materials in the productive cycle is a central issue of bioconversions and of the development of circular production strategies. The problem of handling the by-products of dairy industry has been long known: cheese whey (CW), the main by-product of cheese manufacturing, is a highly polluting waste, due to its high biological and chemical oxygen demand, which originates mostly from the residual lactose, but also from appreciable proteins and lipids content (Table 1) [29, 31]. Several strategies have been proposed to exploit these nutrients, reconverting them in added value compounds, often making use of LAB and of their ability of constitutively thrive on lactose. The growth of LAB on CW-based or CW-containing media, has allowed for the obtainment of various industrially relevant compounds, such as bacteriocins, ethanol, organic acids, often with the aid of metabolic engineering [18, 20, 21, 28, 30]. The utilization of CW as a suitable growth substrate, though, often requires preliminary treatments, which can range from complete deproteinization [20], to the supplementation of nutrients, with the addition of substantial amounts of peptone and yeast extract [21], to treatment with proteases or proteolytic microorganisms [32], and can greatly affect the sustainability and viability of the process. In this paper, we have described for the first time the possibility of using a CW-based medium also for the production of recombinant proteins in *L. lactis*. In the attempt of defining an industrially viable process, we have tried to keep the number of preliminary steps on CW prior to the cultures to a minimum, since pH adjustment and supplementation of catalytic amounts, i.e. 0.05%, of yeast extract have proven enough to produce acceptable biomass accumulation of *L. lactis* NZ9000. Using such a medium, it has been possible to also sustain recombinant protein production with the nisin-inducible NICE system. We have used the pNZ4184 vector carrying the gene coding for the sweet protein MNEI. This protein has been selected for its potential industrial application as a sugar replacer, which has motivated several efforts in the past to develop strategies for recombinant production [35]. Its production in *L. lactis,* a GRAS host, could facilitate the use of the protein as a food additive. Our results clearly indicate that the choice of the appropriate codon usage is a fundamental requisite for the correct expression of the protein, contradicting previous reports on the production of brazzein [46], for which *E. coli* optimized gene corresponded to higher production yields. In the case of MNEI, when the *E. coli* optimized gene was used, protein production was completely abated in the CW-based medium. This result could be related to the specific algorithm used for the *E. coli* codon usage optimization. The coding sequence was optimized to always use the same triplet for each codon, i.e. the most frequently occurring in the *E. coli* coding sequences. In almost 18% of the MNEI coding sequence, the *E. coli* preference is just the opposite of the *L. lactis* one, thus resulting in a coding sequence in which 18% of the codons are classified as “rare” for *Lactococcus.* Interestingly, *L. lactis* is one of the few microbial species for which a complete tRNAome determination was carried out, although in a growth condition investigated quite different from the two tested in the present work.

The general applicability of the CW-based medium has been demonstrated by producing the recombinant catalytic domain of MMP-9, using the previously described system *L. lactis* NZ9000 *clpP*^−^ *htrA*^−^ carrying the pNZ8148 vector coding for the catalytic domain of MMP-9 [44, 45]. Also in this case, recombinant protein production was comparable to the expression in standard rich medium. All the experiments described in this paper have been conducted in a laboratory set up, applying minimal control on the growth parameters, therefore with sub-optimal efficiency. The cultures on ac-CW-based medium always produced lower biomass than the corresponding control cultures in the standard medium. Even so, the yield of recombinant MNEI per liter of culture medium was comparable in the two conditions, due to the observation of higher titers of the target protein in the total protein extract. We can forecast that the introduction of controlled oxygenation and pH alone will compensate for the decrease in biomass accumulation. The production of organic acids throughout the fermentation, coupled to the lack of buffering capability of CW, causes in fact a quick drop in the culture pH to lethal values, which halts biomass accumulation. Moreover, ac-CW medium, as proposed in this paper, contains an unbalanced carbohydrates to proteins ratio that could be reduced by slight modification of the medium formulation. Optimization of these parameters and the use of fermenters will allow greater recoveries both in biomass and recombinant proteins.

## Conclusions

We have presented a new strategy for the production of two recombinant proteins in two strains of *L. lactis* on a growth medium based solely on dairy by-products. Through minimal manipulation and the addition of minimal quantities of yeast extract, it has been possible to sustain microbial growth and to induce protein production levels comparable, if not superior, to the production in rich medium. The simplicity of the process makes it suitable for the application on the industrial scale and prone to wide margins of improvement through the control of the growth parameters.

## Methods

### Bacterial strains and plasmids

*Lactococcus lactis* NZ9000 (*pepN::nisRnisK*) (NIZO) and the double mutant *L. lactis* subsp*. cremoris* NZ9000 *clpP*^−^ *htrA*^−^ [44, 45, 49] used in this study were maintained as frozen glycerol stocks at − 80 °C. The Cm^R^ pNZ8148 plasmid (NIZO), under *nis*A promoter control, was used in this work. MNEI was expressed in *L. lactis* NZ9000, while the catalytic domain of metalloproteinase 9 (MMP-9), from *Bos taurus*, was produced, with a 6×His-tag, in *L. lactis* NZ9000 *clpP*^−^ *htrA*^−^ (*clpP*-*htrA*; erythromycin resistant (Em^R^)) (kindly provided by INRA, Jouy-en-Josas, France; patent nº EP1141337B1) [45, 49]. The gene sequences for MNEI with optimized codon usage for *L. lactis* (*MNEI*-*ll*) and *E. coli* (*MNEI*-*ec*) were purchased from Eurofins Genomics and received within commercial vectors. Both synthetic genes contained the *Not*I restriction enzyme site for the screening of the recombinant clones and were cloned into the pNZ8148 expression vector between the *Nco*I and *Hin*dIII restriction sites (Additional file 1: Figure S1). Plasmid isolation from *L. lactis* cells was achieved with the PureYield kit (Promega) after incubation of the cells with 5 mg/mL Lysozyme, 2 h, 37 °C.

### Compositional analysis of CW

Cheese whey (CW) from the production of ricotta was obtained from “Caseificio Le Terre di Don Peppe Diana” and stored at − 20 °C until used. Ac-CW was obtained by adjusting the pH of CW to 6.8, sterilization by autoclave and centrifugation (14,000×*g*, 30′, 4 °C) to remove the precipitate. Protein determination was performed by Bradford assay (Bio-rad). Carbohydrates determination was obtained by the Phenol/Sulfuric Acid assay [50]. Lipid determination was obtained by the Bligh & Dyer extraction, as reported [51].

### Cell cultures

*Lactococcus lactis* was grown in M17 medium supplemented with 0.5% d-glucose (G-M17), in ac-CW and in ac-CW supplemented with 0.05% Yeast extract. Culture were kept static at 30 °C. Typically, 30 mL cultures were performed in 50 mL tubes. For recombinant strains, the growth medium was supplemented with 5 µg/mL chloramphenicol. In general, overnight cultures of *L. lactis* in G-M17 were diluted into 30 mL of culture medium to an OD600 of 0.1 Bacterial growth rates in G-M17 and in ricotta cheese whey were measured by plating appropriate dilutions of the culture suspension on G-M17 agar plates and counting the CFU after incubation at 30 °C for 24 h.

### Production of MNEI and MMP-9 in *L. lactis*

*L. lactis* NZ9000 competent cells were transformed by electroporation with either the pNZ8148-*MNEI*-*ll* or pNZ8148-*MNEI*-*ec* vector [52]. Electroporation was performed with a Gene Pulser from Bio-rad fitted with 2500 V, 200 X and 25 µF in a pre-cooled 2 cm electroporation cuvette. Samples were then incubated for 2 h at 30 °C in 900 µL restorative medium (G-M17 with 20 mM Mg_2_Cl_2_ and 2 mM Ca_2_Cl_2_). The electroporation mix was centrifuged for 10 min at 10,000×*g* at 4 °C and the pellet was resuspended in 100 µL of G-M17 media and plated. Recombinant cells were grown as described in the previous section. Expression of the MNEI gene was induced by administration of 10 ng/mL nisin in the mid-exponential phase, i.e. 2 h into the growth. At 2, 4 or 16 h post induction, 10 mL from the culture were harvested and the cells were pelleted by centrifugation (10,000×*g*, 4 °C, 30′), washed twice with cold PBS, resuspended in 1 mL of 50 mM sodium acetate buffer, pH 5.5, and disrupted by sonication. The MMP-9 recombinant production was carried out in *L. lactis* NZ9000 *clpP*^−^ *htrA*^−^ recombinant with pNZ8148-*MMP*-*9* [44], following the same conditions previously described for MNEI production.

### SDS-PAGE and Western blot

Total protein content of the sonicated fractions was estimated by Bradford assay using BSA as standard. The purified recombinant MNEI from *E. coli* (MW ~ 11 kDa), obtained as described in [53], was used as positive control. Samples of 10 μg total protein extract were loaded on 12% SDS-PAGE and then blotted onto Immobilon-P transfer membrane (EMD, Millipore Corporation, USA). Membranes were incubated with an rabbit anti-Y65R-MNEI antibody [36] (1:200, Primmbiotech, courtesy of Dr. Nunzia Scotti) and subsequently with a HRP-conjugated anti-rabbit antibody (1:50,000, BioFX Laboratories). Detection of the His-tagged MMP-9 expression in *L. lactis clpP*^*−*^ *htrA*^*−*^ was also performed by western blot. The membranes were incubated with anti-poly-His Peroxidase conjugate (1:2000). In both cases the signal was revealed by enhanced chemiluminescence (ECL) kit (Biorad, Clarity™ western ECL Substrate) and recorded on a ChemiDoc™ MP Imaging System (Biorad).

## Additional files

**Additional file 1: Figure S1.** Alignment of the *MNEI-ec* and *MNEI-ll* gene sequences. Restriction sites used for cloning are indicated in blue (NcoI and Hind III). A NotI site (red) was included for plasmid screening.

**Additional file 2: Figure S2.** Effect of the codon usage on recombinant protein production. Western blot (B) of the total protein extract (10 μg) from *L.lactis* NZ9000 carrying the pNZ8148-*MNEI-ec* vector. A: no induction; B: 2 h post-induction; C: 4 h post induction; D: 16 h post induction; E: MNEI, 50 ng; F: MNEI, 200 ng; G: MNEI, 500 ng.

**Additional file 3: Figure S3.** Evolution of the pH during cell culture. Plot of the pH vs time during the growth of *L. lactis* NZ9000 in G-M17 medium (blue curve) and in ac-CW + 0.05% yeast extract (red curve).

**Additional file 4: Table S1.** Composition of the G-M17 medium.

**Additional file 5: Figure S4.** Effect of the codon usage on recombinant protein production in CW-based medium. Western blot (B) of the total protein extract (10 μg) from *L.lactis* NZ9000 carrying the pNZ8148-*MNEI-ec* vector growth on ac-CW + 0.05% yeast extract. A: no induction; B: 2 h post-induction; C: 4 h post induction; D: 16 h post induction; E: MNEI, 50 ng; F: MNEI, 200 ng; G: MNEI, 500 ng.

## Abbreviations

CW: cheese whey
ac-CW: autoclaved/centrifuged cheese whey
NICE: nisin controlled gene expression system
