# Supplementary figures and images for: Getting value from the waste: recombinant production of a sweet protein by Lactococcus lactis grown on cheese whey
Source: Microb Cell Fact. 2018 Aug 15;17:126. doi: 10.1186/s12934-018-0974-z (PMC6094915; doi:10.1186/s12934-018-0974-z)

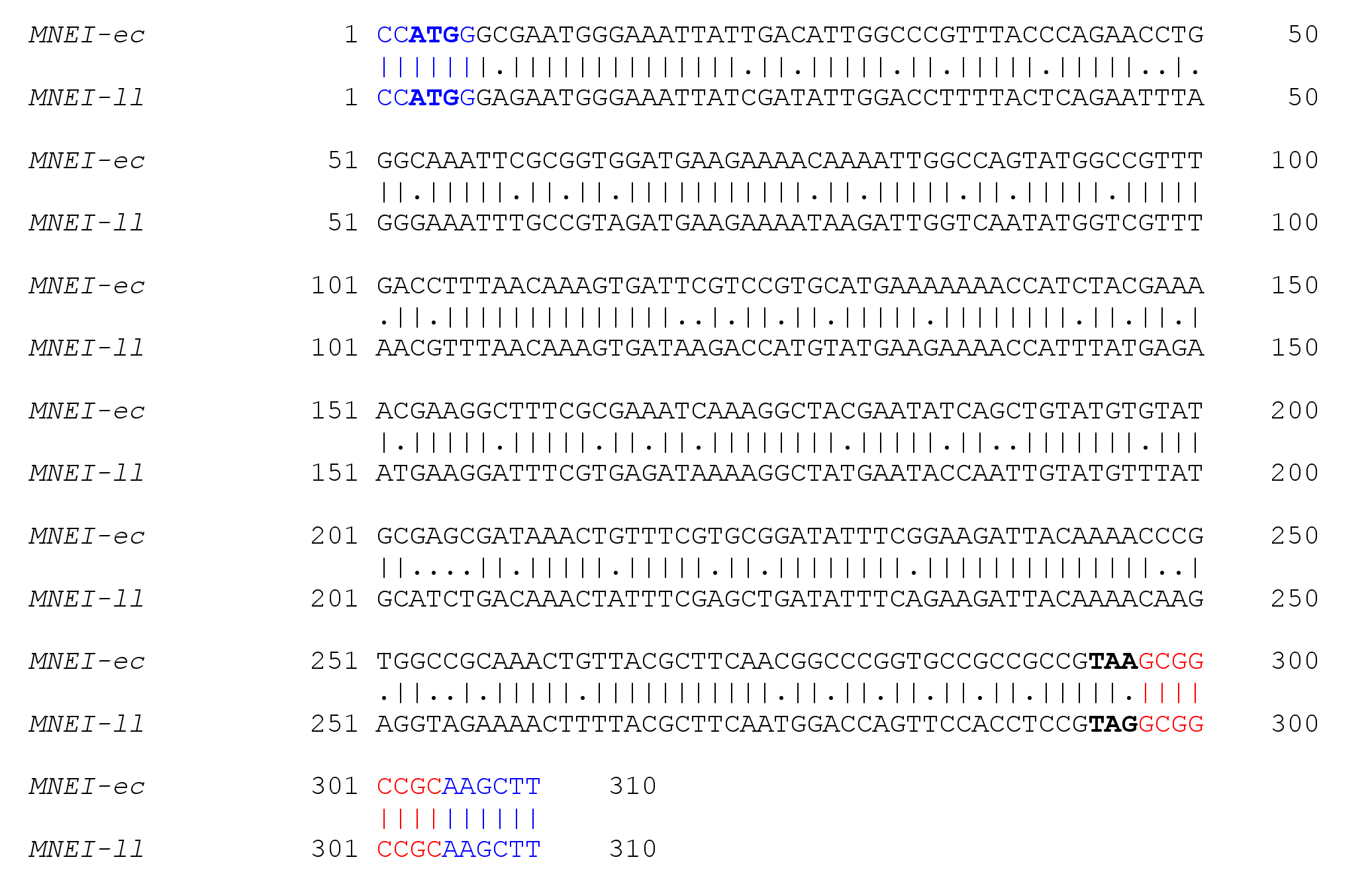

Supplement: Supplementary file 1 — Additional file 1: Figure S1. Alignment of the MNEI-ec and MNEI-ll gene sequences. Restriction sites used for cloning are indicated in blue (NcoI and Hind III). A NotI site (red) was included for plasmid screening. [file 12934_2018_974_MOESM1_ESM.png]

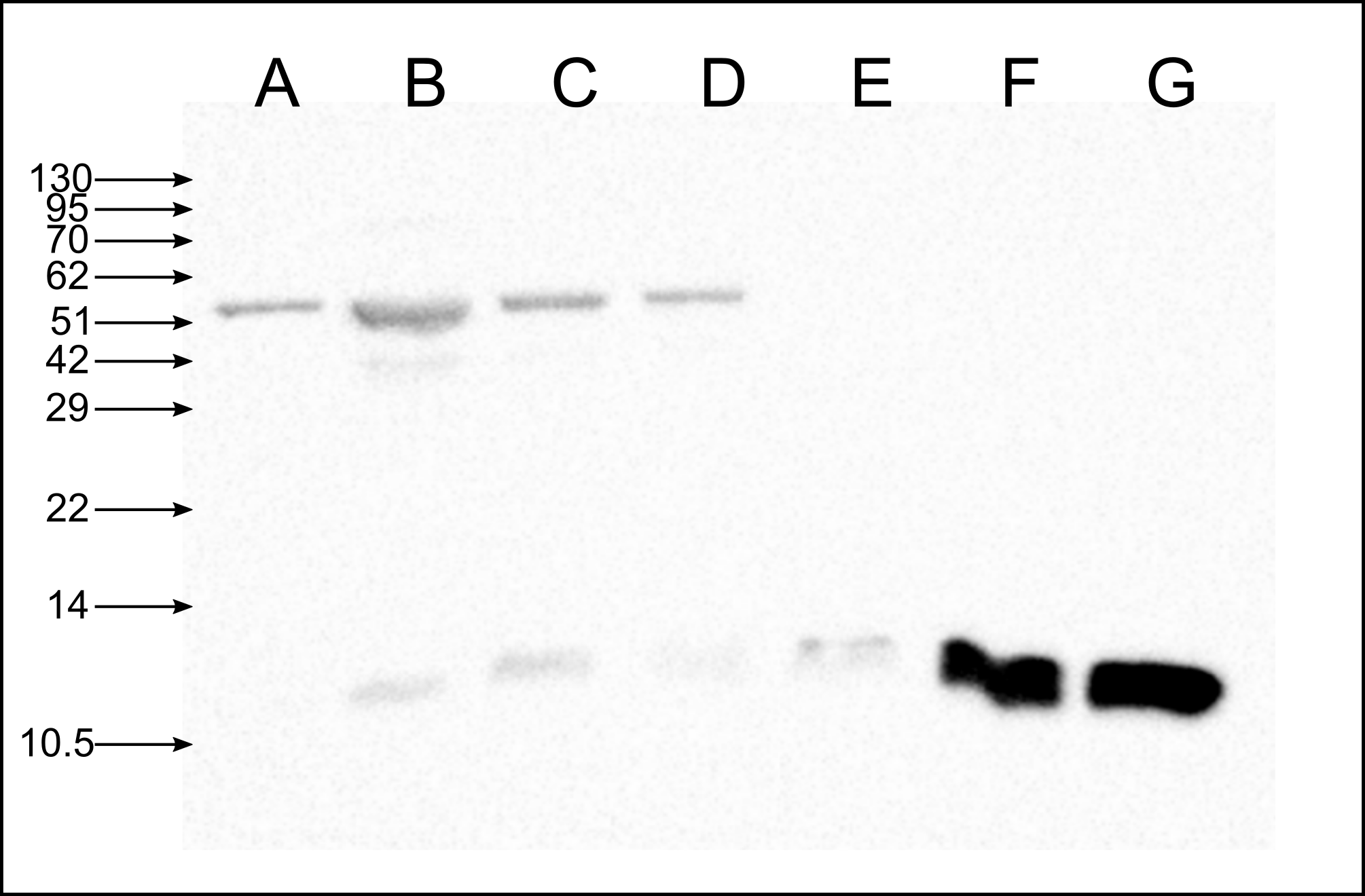

Supplement: Supplementary file 2 — Additional file 2: Figure S2. Effect of the codon usage on recombinant protein production. Western blot (B) of the total protein extract (10 μg) from L.lactis NZ9000 carrying the pNZ8148-MNEI-ec vector. A: no induction; B: 2 h post-induction; C: 4 h post induction; D: 16 h post induction; E: MNEI, 50 ng; F: MNEI, 200 ng; G: MNEI, 500 ng. [file 12934_2018_974_MOESM2_ESM.png]

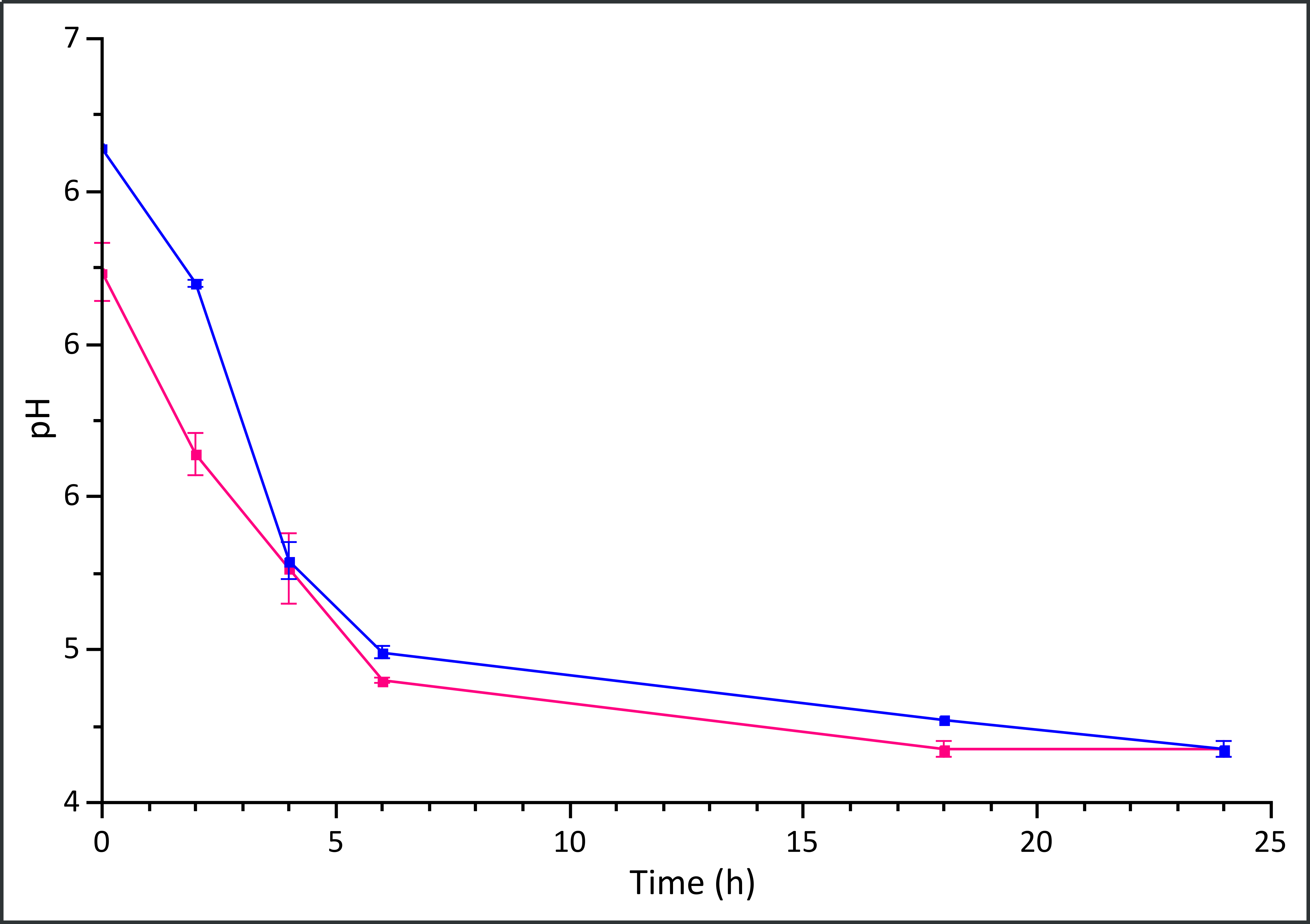

Supplement: Supplementary file 3 — Additional file 3: Figure S3. Evolution of the pH during cell culture. Plot of the pH vs time during the growth of L. lactis NZ9000 in G-M17 medium (blue curve) and in ac-CW + 0.05% yeast extract (red curve). [file 12934_2018_974_MOESM3_ESM.png]

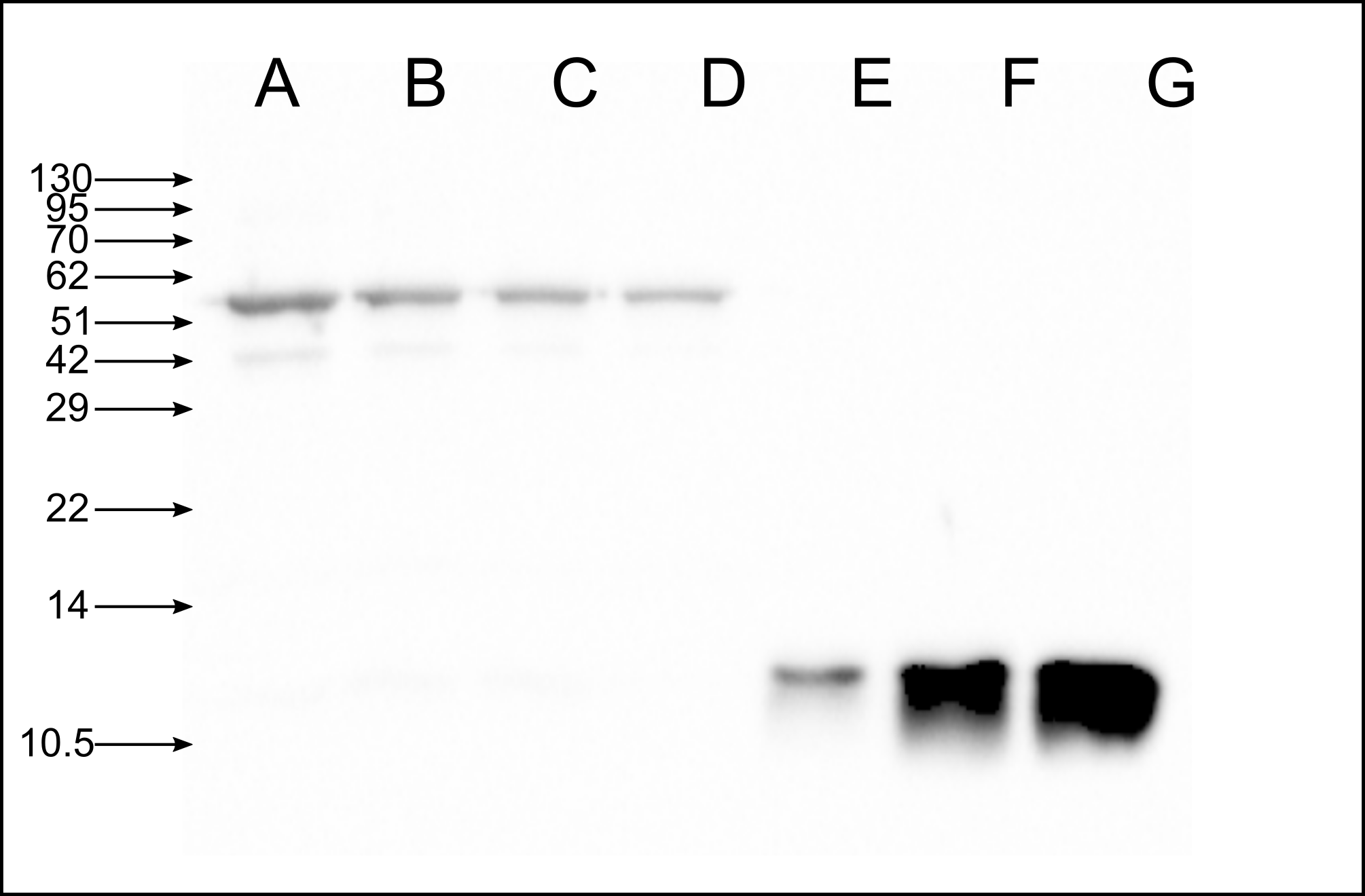

Supplement: Supplementary file 5 — Additional file 5: Figure S4. Effect of the codon usage on recombinant protein production in CW-based medium. Western blot (B) of the total protein extract (10 μg) from L.lactis NZ9000 carrying the pNZ8148-MNEI-ec vector growth on ac-CW + 0.05% yeast extract. A: no induction; B: 2 h post-induction; C: 4 h post induction; D: 16 h post induction; E: MNEI, 50 ng; F: MNEI, 200 ng; G: MNEI, 500 ng. [file 12934_2018_974_MOESM5_ESM.png]
