# Supplementary material for: Getting value from the waste: recombinant production of a sweet protein by Lactococcus lactis grown on cheese whey
Source: Microb Cell Fact. 2018 Aug 15;17:126. doi: 10.1186/s12934-018-0974-z (PMC6094915; doi:10.1186/s12934-018-0974-z)
Supplement: Supplementary file 4 — Additional file 4: Table S1. Composition of the G-M17 medium. [file 12934_2018_974_MOESM4_ESM.docx]

**Table S1: Composition of the G-M17 medium (pH 6.7):**

|  | **Concentration (g/L)** |
| --- | --- |
| Tryptone | 2.5 |
| Meat Peptone | 2.5 |
| Soy Peptone | 5.0 |
| Yeast Extract | 2.5 |
| Meat Extract | 5.0 |
| Sodium glycerophosphate | 19.0 |
| Magnesium sulfate | 0.25 |
| Ascorbic Acid | 0.5 |
| Lactose | 5.0 |
| Glucose | 5.0 |
